# eHealth interventions for chronic pain: Protocol for a systematic review and meta-analysis

**DOI:** 10.1016/j.mex.2025.103475

**Published:** 2025-06-27

**Authors:** Rosario Caruso, Pier Mario Perrone, Karen Barros Parron Fernandes, Cristina Arrigoni, Arianna Magon, Gianluca Conte, Silvia Belloni, Silvana Castaldi

**Affiliations:** a Department of Biomedical Sciences for Health, University of Milan, Milan, Italy; b Clinical Research Service, IRCCS Policlinico San Donato, San Donato Milanese, Italy; c Department of Clinical Sciences and Community Health, University of Milan, Milan, Italy; d Graduate Program of Health Sciences (PPGCS), School of Medicine, Pontifical Catholic University of Parana (PUCPR), Curitiba, Brazil; e School of Medicine, Pontifical Catholic University of Parana (PUCPR), Londrina, Brazil; f Department of Public Health, Experimental and Forensic Medicine, Section of Hygiene, University of Pavia, Pavia, Italy; g Quality Units, Fondazione Ca’ Granda Policlinico di Milano, Milan, Italy

**Keywords:** Chronic pain, ehealth interventions, Telemedicine, Mobile health, Digital health, Network meta-analysis, Pain management, Systematic review, Meta-analysis, eHealth Interventions for Chronic Pain

## Abstract

Chronic pain is a prevalent and costly condition that significantly impairs functional and emotional status as well as quality of life, representing a major cause of incapacity worldwide that requires long-term management strategies.

eHealth interventions, including telemedicine, mobile health applications, and internet-based programs, have emerged as promising approaches to improve pain management by enhancing access to education, psychological support, and self-monitoring tools. However, the efficacy of these interventions remains unclear due to variability in study designs or intervention components, differences in pain conditions (e.g., somatic or neuropathic pain), and outcome measures. This systematic review and meta-analysis aims to synthesize the evidence on the efficacy of eHealth interventions for chronic non-cancer pain in adults, assessing their impact on pain severity, functional outcomes, quality of life, mental health, medication adherence, patient satisfaction, and cost-effectiveness. A network meta-analysis (NMA) will be conducted to compare different eHealth modalities and identify which features contribute most to positive outcomes. Statistical analyses will follow Cochrane guidelines, with the risk of bias and evidence certainty evaluated using the Cochrane RoB 2.0 tool and GRADE framework, respectively. The findings will provide valuable insights for clinicians, policymakers, and researchers, informing best practices for integrating digital health solutions into chronic pain management.

• This systematic review and meta-analysis will assess the efficacy of eHealth interventions for chronic pain management.
• A network meta-analysis will compare different digital health modalities to identify the most effective intervention components.
• The study will evaluate clinical, functional, psychological, adherence, and cost-related outcomes, informing evidence-based practice and policy.

## Graphical abstract

**Figure:**
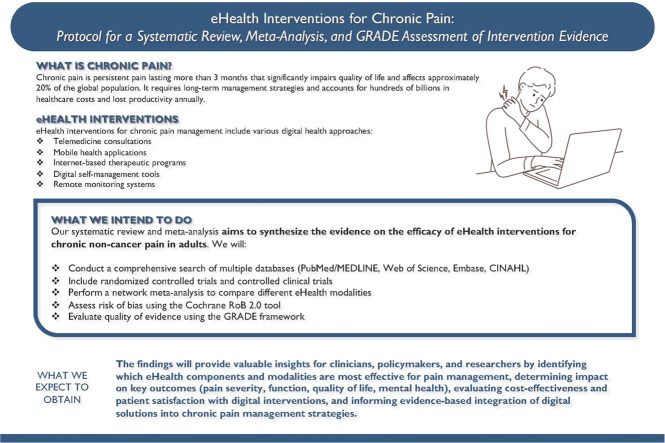

Specifications table

**Table:** 

Subject area:	Medicine and Dentistry
More specific subject area:	Digital Health, Pain Management, Telemedicine, eHealth Interventions, Public Health, Physical Rehabilitation, Nursing, Physiotherapy.
Name of your protocol:	eHealth Interventions for Chronic Pain
Reagents/tools:	Not applicable
Experimental design:	This study will be a systematic review and meta-analysis following PRISMA guidelines. It will include randomized controlled trials (RCTs) and controlled clinical trials assessing the efficacy of eHealth interventions for chronic pain management. A network meta-analysis (NMA) will be conducted to compare different eHealth modalities and intervention components. Statistical analyses will be performed using Stata, and the risk of bias will be assessed using the Cochrane RoB 2.0 tool. The certainty of evidence will be evaluated using the GRADE framework.
Trial registration:	PROSPERO: CRD420251019474
Ethics:	This study is based on previously published data (secondary data) and does not involve human or animal subjects. Ethical approval is, therefore, not required.
Value of the Protocol:	• This systematic review and network meta-analysis will provide a comprehensive synthesis of the evidence on eHealth interventions for chronic pain management. • The study will compare different digital health modalities to identify the most effective intervention components, informing clinical practice and healthcare policy. • Findings will offer insights into patient-centered outcomes, including adherence, satisfaction, and cost-effectiveness, guiding the integration of eHealth into multidisciplinary pain management strategies.

## Background

Chronic pain is a prevalent and costly public health issue, affecting roughly 20 % of the population worldwide and accounting for hundreds of billions in healthcare costs and lost productivity [1,2]. It significantly impairs functional and emotional status as well as quality of life and often requires long-term management strategies [3]. Additionally, inadequate pain control is associated with incapacity, hospital admissions, and a high burden on the public health system [4].

In recent years, electronic health (eHealth) interventions—including telemedicine consultations, mobile health applications, internet-based therapeutic programs, and other digital self-management tools—have emerged as promising approaches to improve chronic pain management outcomes [5]. A growing body of evidence supports the efficacy of eHealth interventions in helping patients manage chronic pain by providing remote access to education, psychological support (e.g., cognitive-behavioral therapy modules), and self-monitoring capabilities [6]. These digital modalities could complement traditional in-person treatments and enhance the reach of pain management services [5,6].

The expansion of telehealth during the COVID-19 pandemic underscored the vital role of eHealth in maintaining continuity of care for chronic conditions [7]. Telemedicine became a lifeline for patients, improving access to care while reducing disparities and promoting health equity when in-person visits were limited [8,9]. Given the rapid growth of various eHealth tools for pain management, there is a pressing need to systematically synthesize the evidence of their efficacy [10].

While individual studies and some reviews suggest that eHealth interventions may yield positive outcomes—such as pain reduction and improved functionality and psychological well-being—a comprehensive, up-to-date systematic review and meta-analysis will clarify the overall efficacy of these interventions across multiple outcomes [3,5,6]. Moreover, eHealth interventions vary in terms of complexity and intensity, ranging from simple educational platforms to fully interactive, therapist-guided programs incorporating multimodal approaches. Given this heterogeneity, a network meta-analysis (NMA) could help compare different eHealth modalities and determine whether certain features (e.g., interactivity level, frequency of patient engagement, integration with healthcare providers) are associated with greater improvements in pain and functional-related outcomes [11]. Such a synthesis is crucial for informing clinicians, physiotherapists, nurses, public health officers, and policymakers about the value of integrating eHealth into multidisciplinary chronic pain management and identifying which aspects of pain (severity, function, quality of life, etc.) are most improved by digital interventions.

This protocol aims to describe the methodological steps required to perform a systematic review to evaluate the efficacy of eHealth interventions in chronic pain management. Specifically, the review will assess the impact of eHealth-based approaches on key outcomes in adults with chronic non-oncological pain, including (a) Reductions in pain severity/intensity (e.g., visual analog scale or numerical rating scale scores) and improvements in physical functional status (primary outcomes); (b) health-related quality of life, psychological outcomes (such as depression or anxiety scores), and cost-related outcomes, including direct medical costs (e.g., healthcare utilization, medication use, hospitalizations), indirect costs (e.g., productivity loss, absenteeism), and cost-effectiveness measures (e.g., cost per quality-adjusted life year [QALY]), which are all secondary outcomes.

## Description of protocol

### Study design

This project will be a systematic review and meta-analysis following Preferred Reporting Items for Systematic Reviews and Meta-Analyses (PRISMA) guidelines, including the NMA extension [12], and the results will be reported according to the same guidelines

### Search strategy and eligibility criteria

A comprehensive literature search will be conducted to capture all relevant studies. We will include trials testing the efficacy of e-health interventions in comparison to a control condition. Randomized controlled trials (blinded or not), non-controlled trials, and cluster-randomized trials will be considered. In cross-over trials, only the first phase of the study will be considered once the ‘carry-over effect’ cannot be excluded and would affect the results from the second phase of the trial. There would be no limitation regarding the follow-up duration, minimal number of participants and country where the trial was conducted.

The following databases will be searched: PubMed/MEDLINE, Web of Science, Embase, CINAHL (Cumulative Index to Nursing and Allied Health Literature), and PsycINFO. Reference lists of included articles will be screened for completeness, and grey literature (e.g., clinical trial registries or conference proceedings) will be considered to minimize publication bias. The search strategy will use a combination of keywords and medical subject headings related to chronic pain (e.g., chronic pain, fibromyalgia, arthritis, low back pain) and eHealth interventions (e.g., telehealth, telemedicine, mHealth, mobile applications, internet-based-intervention digital health, self-management tool).

Searches will be restricted to studies involving human adults and reported in English, Italian, Spanish, and Portuguese. There will be no strict lower date limit, but it is anticipated that most eHealth intervention studies have been published in the last ∼20 years, given technological development (see Table 1). The anticipated upper date limit for the literature search is July 2025; this may be updated if the final search is conducted at a later stage.

**Table 1:** Search strategy (consultation date: 16/06/2025).

**PUBMED** (4218 results)	("Telemedicine"[Mesh] OR “telemedicine”[tw] OR “tele-medicine”[tw] OR “Virtual Medicine”[tw] OR “Tele-Referral”[tw] OR “Tele Referral*”[tw] OR “Mobile Health”[tw] OR “mHealth”[tw] OR “Telehealth”[tw] OR “eHealth”[tw] OR “Telecare”[tw] OR “Tele-Care”[tw] OR “Tele Care”[tw] OR "ehealth interventions"[tw] OR "mobile health intervention*"[tw] OR "digital therap*"[tw] OR "digital health intervention*”[tw] AND "Chronic Pain"[Mesh] OR “Chronic pain”[tw] OR "non-cancer pain"[tw] OR "chronic pain management"[tw]) AND "Randomized Controlled Trial" [Publication Type]
**CINAHL** (4851 results)	((MH Telemedicine+) OR telemedicine OR tele-medicine OR "Virtual Medicine" OR Tele-Referral OR "Tele Referral*" OR "Mobile Health" OR mHealth OR Telehealth OR eHealth OR Telecare OR Tele-Care OR "Tele Care" OR "ehealth interventions" OR "mobile health intervention*" OR "digital therap*" OR "digital health intervention*" AND (MH "Chronic Pain+") OR "Chronic pain" OR "non-cancer pain" OR "chronic pain management") AND (PT "Randomized Controlled Trial")
**WEB OF SCIENCE** (9011 results)	(ALL=Telemedicine OR ALL=tele-medicine OR ALL="Virtual Medicine" OR ALL="Tele Referral*" OR ALL="Mobile Health" OR ALL=mHealth OR ALL=Telehealth OR ALL=eHealth OR ALL=Telecare OR ALL=Tele-Care OR ALL="Tele Care" OR ALL="ehealth interventions" OR ALL="mobile health intervention*" OR ALL="digital therap*" OR ALL="digital health intervention*" AND ALL="Chronic Pain" OR ALL="Chronic pain" OR ALL="non-cancer pain" OR ALL="chronic pain management") AND ALL="Randomized Controlled Trial"
**EMBASE** (360 results)	('telemedicine'/exp OR telemedicine OR 'tele medicine' OR 'virtual medicine' OR 'tele referral' OR 'tele referral*' OR 'mobile health' OR mhealth OR telehealth OR ehealth OR telecare OR 'tele care' OR 'ehealth interventions' OR 'mobile health intervention*' OR 'digital therap*' OR 'digital health intervention*') AND ('chronic pain'/exp OR 'chronic pain' OR 'non-cancer pain' OR 'chronic pain management') AND 'randomized controlled trial'/exp
**PsycINFO** (2778 results)	(exp Telemedicine/ OR telemedicine.mp. OR tele-medicine.mp. OR "Virtual Medicine".mp. OR Tele-Referral.mp. OR "Tele Referral*".mp. OR "Mobile Health".mp. OR mHealth.mp. OR Telehealth.mp. OR eHealth.mp. OR Telecare.mp. OR Tele-Care.mp. OR "Tele Care".mp. OR "ehealth interventions".mp. OR "mobile health intervention*".mp. OR "digital therap*".mp. OR "digital health intervention*".mp. AND exp "Chronic Pain"/ OR "Chronic pain".mp. OR "non-cancer pain".mp. OR "chronic pain management".mp.) AND "Randomized Controlled Trial".pt.

*Note:* The search strategy presented here reflects an advanced preliminary version developed to identify relevant studies on eHealth interventions for chronic pain. Minor modifications may be implemented during the full review process to further optimize the balance between sensitivity and specificity, in alignment with the PRESS 2015 checklist for peer-reviewed electronic search strategies.

The database-specific queries were developed and refined according to the PRESS 2015 guideline to ensure completeness, transparency, and reproducibility of the electronic search strategy [13].

Inclusion criteria will be defined using the Population, Intervention, Comparison, Outcomes, Study Design (PICOS) framework as follows.

• Population: Adults (≥18 years old) with chronic pain (pain duration ≥3 months, any etiology such as musculoskeletal pain, neuropathic pain). Studies focusing on chronic non-cancer pain will be included; cancer-related pain will be excluded, whether pain management will differ from chronic pain management due to its oncological origin, thus requiring specific treatment for the underlying pathology beyond the specific treatment of chronic pain.
• Interventions: eHealth-based interventions aimed at managing or treating chronic pain. This encompasses telemedicine (remote consultations or tele-coaching), internet-delivered programs (web-based cognitive behavioral therapy, online education/support platforms), mobile health applications, wearable digital tools, or other technology-assisted self-management programs. Interventions can be unguided (self-administered digital tools) or guided (involving remote clinician input), delivered via any electronic modality (web, phone, video, apps).
• Comparators: Control conditions may include usual care, standard in-person chronic pain management, wait-list control, attention control, or alternative non-eHealth interventions. Studies must have a comparison group to allow assessment of efficacy (for example, randomized controlled trials comparing an eHealth intervention to standard care).
• Outcomes: Studies must report at least one relevant outcome measure of pain or related domains. Acceptable outcomes include pain severity/intensity (e.g., visual analog scale or numerical rating scale scores), pain interference with function, physical functional status, health-related quality of life, psychological outcomes (such as depression or anxiety scores), patient adherence to or engagement with the intervention, patient satisfaction, or cost-related outcomes. Pain reduction and functional improvement are considered primary outcomes of interest for the meta-analysis. In contrast, outcomes like quality of life, mental health, adherence, and satisfaction are essential secondary outcomes (details on outcomes section).
• Study Design: Randomized controlled trials (RCTs) will be the primary focus for inclusion, as they provide the highest level of evidence for intervention efficacy. If RCT evidence is limited, controlled clinical trials or quasi-experimental studies with comparator groups may be considered, but observational studies without a control group (cohort or case series) will be excluded from the quantitative synthesis.

Studies will be excluded if they: (1) focus on acute pain or perioperative pain management (as opposed to chronic pain) or cancer-related pain due to its management typically involves strategies that differ fundamentally from the approaches used for chronic non-cancer pain; (2) do not involve a digital/eHealth component as a central part of the intervention (e.g., studies of purely in-person interventions, or where technology is used only for data collection and not as the intervention medium); (3) lack a comparison group (for example, single-arm feasibility studies or case series, which cannot inform comparative effectiveness or efficacy); (4) are qualitative studies, reviews, protocols, or editorial/opinion pieces rather than original research on outcomes (though references from reviews will be checked for eligible studies); or (5) are not available in English, Italian, Spanish, and Portuguese, unless a translation is feasible (e.g., if an official non-English version of the article is provided in the HTML format it could be easily translated with web-based translators).

A preliminary categorization of EHealth Interventions for chronic pain management is represented in Table 2. In addition, if multiple publications report on the same patient sample (e.g., a follow-up analysis of an RCT), only the most comprehensive or relevant publication will be included to avoid double-counting data.

**Table 2:** Preliminary categorization of eHealth interventions.

Intervention Category	Description	Delivery Modality	Guidance	Preliminary NMA Classification
Telemedicine	Remote consultations, tele-coaching, virtual follow-ups with healthcare providers.	Phone, video calls, secure messaging platforms.	Guided (clinician involvement)	Telemedicine-based interventions
Internet-Delivered Programs	Web-based cognitive behavioral therapy (CBT), online education/support platforms.	Web-based platforms, online modules, interactive websites.	Guided or unguided (self-directed digital modules)	Internet-based interventions
Mobile Health Applications	Smartphone apps for pain tracking, self-management guidance, biofeedback integration.	Mobile applications (iOS/Android), push notifications, in-app coaching.	Guided or unguided (depends on app features)	Mobile health interventions
Wearable Digital Tools	Wearable sensors or digital tools for pain monitoring, real-time feedback, and intervention delivery.	Smartwatches, biosensors, wearable patches, smart clothing.	Unguided (self-monitoring, AI-based feedback)	Wearable technology interventions
Technology-Assisted Self-Management Programs	Hybrid models using multiple eHealth components for patient self-management and remote clinician guidance.	Multi-platform integration combining apps, web, and teleconsultation features.	Guided (hybrid clinician-patient engagement)	Multimodal eHealth interventions

### Study selection

All search results will be imported into reference management software (Zotero), and duplicates will be removed. The study selection process will follow PRISMA 2020 guidelines for transparent reporting. Prior to starting the formal screening process, reviewers will conduct a calibration phase by independently screening a random sample of 10–20 records to ensure consistent application of the eligibility criteria and refine operational definitions if needed. Discrepancies during calibration will be discussed, and the selection criteria will be clarified or revised accordingly. After calibration, two independent reviewers will screen the titles and abstracts of all retrieved references for potential relevance. Studies that appear to meet inclusion criteria (or where relevance is uncertain) will then be evaluated in full text. Each reviewer will independently assess full-text articles against the predefined inclusion/exclusion criteria. Any disagreements between reviewers regarding eligibility will be resolved through discussion and consensus; a third reviewer will be consulted to adjudicate if disagreements persist. A PRISMA flow diagram will document the study selection process, detailing the number of studies identified, screened, excluded (with reasons for exclusion at the full-text stage), and finally included in the review.

### Data extraction

The review team will develop and pilot-test a standardized data extraction form. For each included study, two reviewers will independently extract key data elements, including study characteristics (author(s), publication year, country/setting, study design, and sample size), population characteristics (mean age, pain condition, pain duration, and relevant comorbidities), intervention details (type of eHealth intervention, core components such as educational modules, psychological support, or self-monitoring features, frequency and intensity of use, duration, level of provider involvement, and degree of interactivity), details of the comparison or control condition (such as usual care, in-person interventions, waitlist control, or alternative digital interventions), and outcomes measured (primary and secondary outcomes with corresponding time points of assessment). Outcome data for all relevant endpoints will be recorded, including baseline and post-intervention pain scores, mean differences between groups, standardized effect sizes, and confidence intervals.

To facilitate an NMA, eHealth interventions will be classified based on their complexity and intensity. Specifically, interventions will be categorized by technology modality (such as telemedicine, mobile health applications, web-based programs, virtual reality, wearable devices, or hybrid models), level of interactivity (ranging from passive interventions with static educational content to highly interactive ones incorporating real-time clinician engagement or peer support features), degree of personalization (generic interventions versus tailored content based on user inputs or clinician guidance), provider involvement (fully automated interventions versus those with clinician oversight), and intensity or duration of use (frequency of patient engagement and length of the intervention period). These classification criteria will enable subgroup analyses and indirect comparisons between different intervention types within the NMA framework.

In cases of missing or incomplete outcome data, we will attempt to contact the corresponding study authors twice to obtain the necessary information. If no response is received, we will try to estimate the missing data using other reported statistics (such as confidence intervals or p-values) [14]. If imputation remains unfeasible, the affected outcomes will be excluded from the meta-analysis but retained in the narrative synthesis. Sensitivity analyses will be conducted to assess the impact of excluding these studies on the overall results.

### Definition of outcomes

Pain reduction and functional improvement are the primary outcomes, as they represent core goals in chronic pain management. Pain reduction refers to changes in pain severity or intensity, as reported by patients using standardized scales such as the Visual Analog Scale (VAS), Numeric Rating Scale (NRS), or validated pain questionnaires [15,16]. This outcome measures the extent to which eHealth interventions alleviate pain symptoms. Functional improvement captures changes in physical functioning and the ability to carry out daily activities despite pain. It may include reductions in pain-related disability, increased physical activity, and improvements in pain interference with work, sleep, and daily tasks. Functional improvement will be assessed (if feasible) using tools such as the Brief Pain Inventory (BPI) interference subscale and other validated functional assessment measures [17].

In addition to pain reduction and functional improvement, eHealth interventions may influence broader aspects of health and healthcare engagement [18]. When available, quality of life will be evaluated in terms of changes in overall well-being or health-related quality of life using instruments such as the Short Form-36 (SF-36), Short-Form 12 (SF-12), EuroQoL-5D (EQ-5D), or World Health Organization Quality of Life (WHOQOL) measures [[19], [20], [21], [22]]. Since chronic pain affects multiple dimensions of life, this outcome will assess whether eHealth interventions contribute to meaningful improvements in overall health and daily living. Mental health outcomes will also be examined, including changes in depression, anxiety, stress, and pain catastrophizing. These could be measured using validated tools such as the Patient Health Questionnaire-9 (PHQ-9) for depression, the Generalized Anxiety Disorder-7 (GAD-7) scale for anxiety, and other relevant psychometric instruments, when used in the primary studies [23,24].

Adherence to treatment will be assessed as an indicator of patient engagement with the intervention and its feasibility in real-world settings. This may include the proportion of patients who complete the intervention, average usage metrics such as the number of logins or sessions attended in a telehealth program, and other adherence indicators. Higher adherence may correlate with improved outcomes and better integration of eHealth into care pathways. Patient satisfaction will be examined as a measure of patient-reported experience and perceived acceptability of the intervention. This will be assessed through satisfaction surveys, Likert-scale ratings, or qualitative feedback on the benefits and usability of eHealth approaches compared to traditional care.

Cost-effectiveness will be evaluated where relevant data are available, considering economic impacts such as healthcare utilization, potential cost savings from reduced travel expenses or emergency room visits, and formal cost-effectiveness analyses. While not all studies provide economic data, any available evidence on the financial feasibility of eHealth interventions will be synthesized.

### Risk of bias assessment

To evaluate the internal validity of the included studies, two reviewers will independently assess the risk of bias in each trial using the Cochrane Collaboration’s Risk of Bias tool (RoB 2.0) [25]. This tool evaluates potential bias across multiple domains, including the randomization process, deviations from intended interventions, missing outcome data, outcome measurement, and selective reporting. Each study will receive an overall risk-of-bias judgment categorized as “Low,” “Some concerns,” or “High” risk of bias. Any disagreements between reviewers will be resolved through discussion, with the involvement of a third reviewer, if consensus cannot be reached. The results of the risk-of-bias assessment will be reported in the review, for example, through a risk-of-bias summary table. Additionally, sensitivity analyses will be conducted to assess the impact of studies classified as having a high risk of bias on the overall findings.

### Data analysis

We will first conduct a qualitative synthesis of findings across studies, describing the range of interventions and outcomes [26]. Where studies are sufficiently homogeneous in terms of population, intervention, comparator, and outcome measures, we will perform a quantitative meta-analysis using appropriate pooling techniques. For continuous outcomes (such as pain severity measured on a numeric scale), we will calculate either weighted mean differences (WMD) or standardized mean differences (SMD) with 95 % confidence intervals, depending on whether studies use the same or different measurement instruments [27]. For dichotomous outcomes (such as a threshold of pain relief achieved or patient satisfaction rated as satisfactory vs. not), we will pool data using risk ratios or odds ratios with 95 % confidence intervals. A random-effects model will be employed, acknowledging the likelihood of variability among studies in interventions and patient populations.

The primary analysis will use available-case data under the missing at random (MAR) assumption [14]. Sensitivity analyses will explore plausible departures from MAR using two strategies: (a) reason-informed imputations or simple single imputation (e.g., best/worst-case assumptions), and (b) uncertainty-based approaches such as pattern-mixture models with informative missingness parameters, as described by Mavridis and White [14]. We will also conduct sensitivity analyses to assess the impact of excluding studies with unresolvable missing data on overall conclusions.

We will assess statistical heterogeneity using the Chi-square test (Cochran Q) and the I² statistic; an I² value above 50 % will be considered indicative of substantial heterogeneity. If high heterogeneity is detected, we will explore potential sources via subgroup analyses or meta-regression, contingent on data availability. Subgroup analyses will be used for qualitatively defined factors (e.g., comparing different eHealth modalities such as telemedicine vs. mobile apps or different pain conditions), while meta-regression will be considered for quantitatively measured moderators (e.g., intervention duration, frequency of use). Sensitivity analyses (for instance, excluding studies at high risk of bias) will also be conducted to evaluate the robustness of the findings.

If the data structure and availability permit, we will perform an NMA to compare multiple eHealth interventions across a common comparator. This approach will allow for indirect comparisons between different eHealth modalities and facilitate the evaluation of which specific intervention components or features (e.g., level of interactivity, intensity, or provider involvement) are most effective. Ranked forest plots will be used to show the results of the NMA, in which the relative efficacy of the interventions, as well as the precision of the estimates, are presented. Moreover, we will test the transitivity and potential inconsistency of the model to verify its feasibility. All statistical analyses will be conducted in Stata Statistical Software: Release 18. (College Station, TX: StataCorp LLC) [28]. Each outcome will be pooled separately (no aggregation of different outcome types), and results will be presented in forest plots. Publication bias will be assessed through funnel plots for the primary outcomes if ten or more studies are available, and Egger’s test will be used to detect potential asymmetry. If evidence of publication bias emerges, we will discuss its possible impact on the results. When the heterogeneity of outcome measures or insufficient data preclude meta-analysis for certain outcomes, we will either use meta-regression and subgroup analyses to explore the sources of heterogeneity, or we will provide a narrative synthesis, describing the direction and magnitude of effects in each study and explaining any discrepancies among findings. Throughout the data analysis, we will adhere to PRISMA reporting standards to ensure transparency and reproducibility.

To further assess the robustness of statistically significant findings, we will perform Trial Sequential Analysis (TSA) for the primary outcomes (i.e., pain reduction and functional improvement), provided sufficient data are available [29]. TSA will be used to evaluate whether the cumulative evidence is sufficient to draw firm conclusions by applying monitoring boundaries for benefit, harm, and futility, and by estimating the required information size. Analyses will be conducted using the TSA Computer program, version 0.9.5.10 Beta (The Copenhagen Trial Unit, Centre for Clinical Intervention Research, The Capital Region, Copenhagen University Hospital – Rigshospitalet, 2021). This approach will help control for the risk of random errors due to sparse data and repeated significance testing in cumulative meta-analyses.

### Subgroup analysis

eHealth interventions will be categorized according to their complexity and intensity, ensuring a structured comparison of different modalities. These classifications will allow for subgroup analyses and indirect comparisons to identify which intervention features contribute most effectively to pain management outcomes (see Table 2). Subgroup analyses will be performed to assess the impact of specific eHealth intervention characteristics on treatment efficacy. Technology modality will serve as a fundamental categorization, distinguishing telemedicine, mobile health applications, web-based programs, virtual reality interventions, wearable devices, and hybrid models. Given that these modalities differ in their accessibility, engagement mechanisms, and integration within healthcare systems, their relative effectiveness in reducing pain severity and improving functional and psychological outcomes will be compared within the NMA framework.

The level of interactivity will be considered a key moderator, distinguishing between passive interventions that deliver static educational content and highly interactive programs incorporating real-time clinician engagement, gamification, or peer support features. Higher interactivity is hypothesized to be associated with greater adherence and improved treatment outcomes. The extent to which interactivity enhances pain relief and quality of life will be examined through subgroup analyses comparing interventions with minimal engagement to those providing dynamic, user-responsive features.

Another crucial factor will be the degree of personalization, where interventions will be categorized as either generic or tailored based on user inputs or clinician guidance. Personalized interventions may yield superior outcomes by adapting to patient-specific needs and behavioral patterns, thereby improving adherence and self-efficacy. To test this hypothesis, subgroup analyses will compare standardized interventions against those incorporating adaptive algorithms or individualized content.

Provider involvement will be assessed to differentiate between fully automated interventions and those incorporating clinician oversight. Digital interventions that include remote professional support may offer additional benefits, such as enhanced patient motivation and improved adherence. Subgroup analyses will explore whether clinician-guided interventions lead to significantly greater improvements in pain and functional outcomes compared to self-directed, fully automated programs.

Finally, intervention intensity and duration of use will be evaluated to determine the relationship with treatment efficacy. Interventions will be categorized as short-term (<4 weeks), medium-term (4–12 weeks), or long-term (>12 weeks) based on the duration of active use. Frequency of engagement (e.g., daily vs. weekly use) will also be recorded. Subgroup analyses will assess whether higher intensity and longer duration of use are associated with better outcomes, or whether treatment effects tend to plateau over time due to reduced engagement or user fatigue. Additionally, we will explore whether patient adherence to the intervention and levels of satisfaction act as moderators of treatment efficacy.

### GRADE assessment of intervention evidence

To evaluate the overall quality and strength of the evidence for eHealth interventions in chronic pain management, we will apply the Grading of Recommendations, Assessment, Development, and Evaluations (GRADE) framework [[30], [31], [32]]. This approach systematically assesses the certainty of evidence for each primary and secondary outcome based on five key domains: risk of bias, inconsistency, indirectness, imprecision, and publication bias.

Each outcome will be assigned an initial rating based on the study design, with RCTs considered high-certainty evidence. The certainty may then be downgraded if significant concerns arise in any of the five GRADE domains. Studies at high risk of bias (e.g., inadequate randomization or blinding) may lower the overall confidence in the findings. Inconsistency will be assessed based on heterogeneity across studies, and indirectness will be evaluated by considering whether the included studies directly address the research question. Imprecision will be determined by examining the confidence intervals and the total number of events included in the analysis, while publication bias will be explored through funnel plots and statistical tests such as Egger’s test.

The final GRADE assessment will classify the quality of evidence for each outcome into four levels: high, moderate, low, or very low certainty. High-certainty evidence suggests that further research is unlikely to change confidence in the estimated effect, while very low-certainty evidence indicates a high degree of uncertainty in the findings.

GRADE Summary of Findings (SoF) tables will be prepared to present the certainty of evidence for key outcomes, including pain reduction, functional improvement, quality of life, mental health outcomes, adherence, patient satisfaction, and cost-effectiveness. These tables will help clinicians, policymakers, and healthcare providers interpret the strength of the evidence supporting eHealth interventions in chronic pain management and guide recommendations for their integration into clinical practice.

### Protocol validation

Previous narrative and scoping reviews have provided encouraging insights into the potential of eHealth interventions for chronic pain management, highlighting their role in improving patient access to care, self-management strategies, and psychological well-being [5,6]. This review aims to expand upon existing evidence by employing a more nuanced classification of eHealth interventions, distinguishing intervention types based on factors such as interactivity level, clinician involvement, and intervention intensity. Additionally, it will assess the role of different healthcare providers in delivering eHealth interventions, differentiating between clinician-guided, self-administered, and multidisciplinary approaches. While prior systematic reviews and protocols have explored similar questions in chronic non-cancer pain populations, there remains a need to refine our understanding of how specific intervention characteristics influence patient outcomes by applying the GRADE approach to the emerging evidence [33,34].

However, this systematic review and meta-analysis will introduce several key novelties. First, it will employ (if feasible) an NMA approach, allowing for indirect comparisons between different eHealth modalities and identifying which intervention features—such as interactivity level, intensity, or provider involvement—are most effective. This will enable a more nuanced understanding of the heterogeneity of eHealth interventions, moving beyond binary comparisons of digital versus standard care. Second, this study will assess a comprehensive set of outcomes spanning clinical, functional, psychological, and economic dimensions, providing a more holistic evaluation of the impact of eHealth interventions. Including adherence and patient satisfaction as core outcomes will offer insight into the feasibility and acceptability of digital health tools, which are crucial for real-world implementation. Furthermore, by examining cost-related outcomes if retrieved from primary studies, this review will contribute to discussions on the economic sustainability of eHealth interventions, which is particularly relevant given the increasing burden of chronic pain on healthcare systems.

From a public health perspective, while recent priorities have focused on large-scale initiatives such as vaccination campaigns and pandemic preparedness [[35], [36], [37]], chronic disease management remains a critical challenge [[38], [39], [40]]. Chronic pain affects millions globally, contributing to disability, reduced workforce participation, and escalating healthcare costs [[40], [41], [42], [43], [44]]. This study aims to provide evidence-based guidance for integrating digital health solutions into multidisciplinary pain management models, ensuring that advances in eHealth technology translate into tangible benefits for patient care and for the sustainability of health care systems.

## Limitations

This protocol has several potential limitations that should be acknowledged. First, the inclusion of only studies published in Italian, Spanish, and Portuguese, unless a translation is feasible, may introduce language bias, potentially omitting relevant findings.

Second, while NMA will allow for indirect comparisons between different eHealth interventions, its validity depends on assumption homogeneity and transitivity, meaning that included studies must have comparable populations, interventions, and outcomes. If substantial heterogeneity is present, the ability to draw robust conclusions from the NMA may be limited, and results will need to be interpreted with caution.

Third, variability in intervention design, intensity, and delivery across studies may pose challenges in standardizing classifications for subgroup analyses. Differences in technological platforms, levels of interactivity, or clinician involvement may make direct comparisons more complex. While we will attempt to categorize interventions based on predefined criteria, some overlap or inconsistencies may remain.

Fourth, self-reported outcome measures (such as pain intensity, quality of life, and adherence) may introduce subjectivity and recall bias, which could affect the reliability of effect estimates. Additionally, follow-up durations may vary across studies, with some trials only reporting short-term effects of eHealth interventions. As chronic pain management often requires long-term solutions, findings may not fully capture the sustainability of intervention benefits.

Lastly, publication bias remains a concern, particularly if smaller studies with null results are underreported. We will attempt to assess this through funnel plot analysis and statistical tests, but the possibility of selective publication cannot be entirely ruled out.

Despite these limitations, this systematic review and meta-analysis will provide a comprehensive synthesis of the current evidence on eHealth interventions for chronic pain and will generate valuable insights into their effectiveness, feasibility, and impact on patient outcomes.

## CRediT authorship contribution statement

**Rosario Caruso:** Conceptualization, Methodology, Supervision, Writing – original draft. **Pier Mario Perrone:** Data curation, Formal analysis, Writing – original draft. **Karen Barros Parron Fernandes:** Supervision, Validation, Writing – review & editing. **Cristina Arrigoni:** Methodology, Supervision, Writing – review & editing. **Arianna Magon:** Software, Data curation, Project administration, Writing – review & editing. **Gianluca Conte:** Visualization, Validation, Writing – review & editing. **Silvia Belloni:** Writing – original draft, Conceptualization, Methodology, Writing – review & editing. **Silvana Castaldi:** Supervision, Project administration, Writing – review & editing.

## Declaration of competing interest

The authors declare that they have no known competing financial interests or personal relationships that could have appeared to influence the work reported in this paper.

## Data Availability

Protocol Article.

## References

[1] Liikkanen S., Mäkinen M., Huttunen T., Sarapohja T., Stenfors C., Eccleston C. (2022). Body movement as a biomarker for use in chronic pain rehabilitation: an embedded analysis of an RCT of a virtual reality solution for adults with chronic pain. Front. Pain Res..

[2] Hadjiat Y., Arendt-Nielsen L. (2023). Digital health in pain assessment, diagnosis, and management: overview and perspectives. Front. Pain Res..

[3] Maheta B., Kraft A., Interrante N., Fereydooni S., Bailenson J., Beams B., Keny C., Osborne T., Giannitrapani K., Lorenz K. (2025). Using virtual reality to improve outcomes related to quality of life among older adults with serious illnesses: systematic review of randomized controlled trials. J. Med. Internet Res..

[4] Domenichiello A.F., Ramsden C.E. (2019). The silent epidemic of chronic pain in older adults. Prog. Neuro Psychopharmacol. Biol. Psychiatry.

[5] Bartels S.L., Pelika A., Taygar A.S., Wicksell R.K. (2025). Digital approaches to chronic pain: a brief meta-review of eHealth interventions - current evidence and future directions. Curr. Opin. Psychol..

[6] Weatherly S., McKenna T., Wahba S., Friedman A., Goltry W., Wahid T., Abourahma H., Lee K., Rehman A., Odeh A., Costin J. (2024). Effectiveness of digital health interventions (DHI) in Chronic pain management: a scoping review of current evidence and emerging trends. Cureus.

[7] Biagioli V., Albanesi B., Belloni S., Piredda A., Caruso R. (2021). Living with cancer in the COVID-19 pandemic: an Italian survey on self-isolation at home. Eur. J. Cancer Care.

[8] Cerfoglio S., Capodaglio P., Rossi P., Verme F., Boldini G., Cvetkova V., Ruggeri G., Galli M., Cimolin V. (2023). Tele-rehabilitation interventions for motor symptoms in COVID-19 patients: a narrative review. Bioengineering.

[9] Çelik Z., Kafa N., Güzel N.A., Köktürk N. (2024). The effects of physical activity tele-counseling intervention on physical activity, functional performance, and quality of life in post-COVID-19 conditions: a randomized controlled trial. Expert Rev. Respir. Med..

[10] Latif-Zade T., Tucci B., Verbovetskaya D., Bialkin E., Ng B., Heddon S., Berteau J.-P. (2021). Systematic review shows tele-rehabilitation might achieve comparable results to office-based rehabilitation for decreasing pain in patients with knee osteoarthritis. Medicina (B Aires).

[11] White I.R. (2015). Network meta-analysis. Stata J. Promot. Commun. Stat. Stata.

[12] Page M.J., McKenzie J.E., Bossuyt P.M., Boutron I., Hoffmann T.C., Mulrow C.D., Shamseer L., Tetzlaff J.M., Akl E.A., Brennan S.E., Chou R., Glanville J., Grimshaw J.M., Hróbjartsson A., Lalu M.M., Li T., Loder E.W., Mayo-Wilson E., McDonald S., McGuinness L.A., Stewart L.A., Thomas J., Tricco A.C., Welch V.A., Whiting P., Moher D. (2021). The PRISMA 2020 statement: an updated guideline for reporting systematic reviews. BMJ.

[13] McGowan J., Sampson M., Salzwedel D.M., Cogo E., Foerster V., Lefebvre C. (2016). PRESS Peer Review of Electronic search strategies: 2015 guideline statement. J. Clin. Epidemiol..

[14] Mavridis D., White I.R. (2020). Dealing with missing outcome data in meta-analysis. Res. Synth. Methods.

[15] Hawker G.A., Mian S., Kendzerska T., French M. (2011). Measures of adult pain: visual Analog scale for Pain (VAS Pain), numeric rating scale for Pain (NRS Pain), McGill Pain questionnaire (MPQ), short-form McGill Pain questionnaire (SF-MPQ), chronic Pain grade scale (CPGS), short form-36 bodily pain scale (SF-36 BPS), and measure of intermittent and constant osteoarthritis pain (ICOAP). Arthritis Care Res..

[16] Thong I.S.K., Jensen M.P., Miró J., Tan G. (2018). The validity of pain intensity measures: what do the NRS, VAS, VRS, and FPS-R measure?. Scand. J. Pain.

[17] Bonafé F.S.S., de Campos L.A., Marôco J., Campos J.A.D.B. (2019). Brief Pain Inventory: a proposal to extend its clinical application. Eur. J. Pain.

[18] Trimarchi L., Caruso R., Magon G., Odone A., Arrigoni C. (2021). Clinical pathways and patient-related outcomes in hospital-based settings: a systematic review and meta-analysis of randomized controlled trials. Acta Biomed..

[19] Dempster M., Donnelly M. (2000). Selecting a measure of health related quality of life. Soc. Work Health Care.

[20] Hays R.D., Hahn H., Marshall G. (2002). Use of the SF-36 and other health-related quality of life measures to assess persons with disabilities. Arch. Phys. Med. Rehabil..

[21] Carr A.J., Thompson P.W., Kirwan J.R. (1996). Quality of life measures. Br. J. Rheumatol..

[22] Ware J.E. (1976). Scales for measuring general health perceptions. Health Serv. Res..

[23] Spitzer R.L., Kroenke K., Williams J.B.W., Löwe B. (2006). A brief measure for assessing generalized anxiety disorder: the GAD-7. Arch. Intern. Med..

[24] Levis B., Benedetti A., Thombs B.D. (2019). DEPRESsion screening data (DEPRESSD) collaboration, accuracy of Patient health questionnaire-9 (PHQ-9) for screening to detect major depression: individual participant data meta-analysis. BMJ.

[25] Sterne J.A.C., Savović J., Page M.J., Elbers R.G., Blencowe N.S., Boutron I., Cates C.J., Cheng H.-Y., Corbett M.S., Eldridge S.M., Emberson J.R., Hernán M.A., Hopewell S., Hróbjartsson A., Junqueira D.R., Jüni P., Kirkham J.J., Lasserson T., Li T., McAleenan A., Reeves B.C., Shepperd S., Shrier I., Stewart L.A., Tilling K., White I.R., Whiting P.F., Higgins J.P.T. (2019). RoB 2: a revised tool for assessing risk of bias in randomised trials. BMJ.

[26] Deeks J.J., Higgins J.P., Altman D.G., Higgins J.P.T., Thomas J., Chandler J., Cumpston M., Li T., Page M.J., Welch V.A. (2019). Cochrane Handbook for Systematic Reviews of Interventions.

[27] Belloni S., Arrigoni C., Arcidiacono M.A., Baroni I., Conte G., Dellafiore F., Ghizzardi G., Magon A., Villa G., Caruso R. (2023). A systematic review of systematic reviews and pooled meta-analysis on psychosocial interventions for improving cancer-related fatigue. Semin. Oncol. Nurs..

[28] Almquist Y.B., Åkesson C., Brännström L. (2021). An applied guide to quantitative methods with Stata. Res. Rep. Public Health Sci..

[29] Clephas P.R.D., Kranke P., Heesen M. (2023). How to perform and write a trial sequential analysis. Anaesthesia.

[30] Guyatt G.H., Oxman A.D., Vist G.E., Kunz R., Falck-Ytter Y., Alonso-Coello P., Schünemann H.J. (2008). GRADE: an emerging consensus on rating quality of evidence and strength of recommendations. BMJ.

[31] Balshem H., Helfand M., Schünemann H.J., Oxman A.D., Kunz R., Brozek J., Vist G.E., Falck-Ytter Y., Meerpohl J., Norris S., Guyatt G.H. (2011). GRADE guidelines: 3. Rating the quality of evidence. J. Clin. Epidemiol..

[32] Guyatt G., Oxman A.D., Sultan S., Brozek J., Glasziou P., Alonso-Coello P., Atkins D., Kunz R., Montori V., Jaeschke R., Rind D., Dahm P., Akl E.A., Meerpohl J., Vist G., Berliner E., Norris S., Falck-Ytter Y., Schünemann H.J. (2013). GRADE guidelines: 11. Making an overall rating of confidence in effect estimates for a single outcome and for all outcomes. J. Clin. Epidemiol..

[33] Slattery B.W., Haugh S., O’Connor L., Francis K., Dwyer C.P., O’Higgins S., Egan J., McGuire B.E. (2019). An evaluation of the effectiveness of the modalities used to deliver electronic health interventions for chronic pain: systematic review with network meta-analysis. J. Med. Internet Res..

[34] Slattery B.W., Haugh S., Francis K., O’Connor L., Barrett K., Dwyer C.P., O’Higgins S., Egan J., McGuire B.E. (2017). Protocol for a systematic review with network meta-analysis of the modalities used to deliver eHealth interventions for chronic pain. Syst. Rev..

[35] Lecce M., Perrone P.M., Bonalumi F., Castaldi S., Cremonesi M. (2021). 2020-21 influenza vaccination campaign strategy as a model for the third COVID-19 vaccine dose?. Acta Biomed. Atenei Parm..

[36] Biganzoli G., Mendola M., Perrone P.M., Antonangeli L.M., Longo A.B.E., Carrer P., Colosio C., Consonni D., Marano G., Boracchi P., Biganzoli E., Castaldi S. (2024). The effectiveness of the third dose of COVID-19 vaccine: when should it be performed?. Vaccines.

[37] Castaldi S., Perrone P.M., Luconi E., Marano G., Auxilia F., Maraschini A., Bono P., Alagna L., Palomba E., Bandera A., Boracchi P., Biganzoli E. (2022). Hospital acquired infections in COVID-19 patients in sub intensive care unit: analysis of two waves of admissions. Acta Biomed. Atenei Parm..

[38] Vlaeyen J.W.S., Maher C.G., Wiech K., Van Zundert J., Meloto C.B., Diatchenko L., Battié M.C., Goossens M., Koes B., Linton S.J. (2018). Low back pain. Nat. Rev. Dis. Primers.

[39] Weisman S.M., Ciavarra G., Cooper G. (2024). What a pain in the … back: a review of current treatment options with a focus on naproxen sodium. J. Pharm. Pharm. Sci..

[40] Manniche C., Hall G.M. (2021). Chronic low back pain, modic changes and low-grade virulent infection: efficacy of antibiotic treatment. Future Sci. OA.

[41] Miniati M., Orrù G., Paroli M., Cinque M., Paolicchi A., Gemignani A., Perugi G., Ciacchini R., Marazziti D., Palagini L., Conversano C. (2023). Patients with chronic pain: are mindfulness traits protective against distress, Anxiety and depression?. Clin. NeuroPsychiatry.

[42] Goldberg D.S., McGee S.J. (2011). Pain as a global public health priority. BMC Public Health.

[43] Sommer C., Rittner H. (2024). Pain research in 2023: towards understanding chronic pain. Lancet Neurol..

[44] Zhu M., Zhang J., Liang D., Qiu J., Fu Y., Zeng Z., Han J., Zheng J., Lin L. (2024). Global and regional trends and projections of chronic pain from 1990 to 2035: analyses based on global burden of diseases study 2019. Br. J. Pain.

